# Temporal Proteomic Analysis of BK Polyomavirus Infection Reveals Virus-Induced G_2_ Arrest and Highly Effective Evasion of Innate Immune Sensing

**DOI:** 10.1128/JVI.00595-19

**Published:** 2019-07-30

**Authors:** Laura G. Caller, Colin T. R. Davies, Robin Antrobus, Paul J. Lehner, Michael P. Weekes, Colin M. Crump

**Affiliations:** a Division of Virology, Department of Pathology, University of Cambridge, Cambridge, United Kingdom; b Cambridge Institute for Medical Research, Addenbrooke’s Hospital, Cambridge, United Kingdom; International Centre for Genetic Engineering and Biotechnology

**Keywords:** cell cycle, immune evasion, innate immunity, polyomavirus, proteomics

## Abstract

BK polyomavirus (BKPyV) is a small DNA virus that establishes a life-long persistent infection in the urinary tract of most people. BKPyV is known to cause severe morbidity in renal transplant recipients and can lead to graft rejection. The simple 5.2-kbp double-stranded DNA (dsDNA) genome expresses just seven known proteins; thus, it relies heavily on the host machinery to replicate. How the host proteome changes over the course of infection is key to understanding this host-virus interplay. Here, for the first time quantitative temporal viromics has been used to quantify global changes in >9,000 host proteins in two types of primary human epithelial cells throughout 72 h of BKPyV infection. These data demonstrate the importance of cell cycle progression and pseudo-G_2_ arrest in effective BKPyV replication, along with a surprising lack of an innate immune response throughout the whole virus replication cycle. BKPyV thus evades pathogen recognition to prevent activation of innate immune responses in a sophisticated manner.

**IMPORTANCE** BK polyomavirus can cause serious problems in immune-suppressed patients, in particular, kidney transplant recipients who can develop polyomavirus-associated kidney disease. In this work, we have used advanced proteomics techniques to determine the changes to protein expression caused by infection of two independent primary cell types of the human urinary tract (kidney and bladder) throughout the replication cycle of this virus. Our findings have uncovered new details of a specific form of cell cycle arrest caused by this virus, and, importantly, we have identified that this virus has a remarkable ability to evade detection by host cell defense systems. In addition, our data provide an important resource for the future study of kidney epithelial cells and their infection by urinary tract pathogens.

## Abstract (precis)

BK polyomavirus can cause serious problems in immune-suppressed patients, in particular, kidney transplant recipients who can develop polyomavirus-associated kidney disease. In this work, we have used advanced proteomics techniques to determine the changes to protein expression caused by infection of two independent primary cell types of the human urinary tract (kidney and bladder) throughout the replication cycle of this virus. Our findings have uncovered new details of a specific form of cell cycle arrest caused by this virus, and, importantly, we have identified that this virus has a remarkable ability to evade detection by host cell defense systems. In addition, our data provide an important resource for the future study of kidney epithelial cells and their infection by urinary tract pathogens.

## INTRODUCTION

BK polyomavirus (BKPyV) is a small, nonenveloped, double-stranded DNA (dsDNA) virus that was first identified in 1971 (1). As a ubiquitous pathogen, it establishes a life-long persistent infection in the kidneys of most humans (2). While infection with BKPyV is subclinical in the vast majority of individuals, it is a significant cause of morbidity in the immunosuppressed, in particular, in kidney and hematopoietic stem cell transplant (HSCT) recipients. Polyomavirus-associated nephropathy (PVAN) affects ∼8% of kidney transplant patients; however, treatment is currently limited to a reduction in immune suppression. Only a small number of anti-BKPyV drugs are available, all exhibiting significant nephrotoxicity, leading to graft decline of function and loss in ∼85% of PVAN sufferers (3). In up to 15% of HSCT patients, BKPyV leads to hemorrhagic cystitis (HC) and severely reduced rates of HSCT recovery (4).

As with all polyomaviruses, BKPyV is structurally simple. The dsDNA genome is ∼5.2 kbp long and encodes seven proteins, three of which form the virus capsid (VP1, VP2, and VP3). The four nonstructural proteins (large T antigen [LTAg], small T antigen [stAg], truncTAg, and agnoprotein) have numerous functions and interact with multiple host factors. For example, LTAg binds members of the retinoblastoma (Rb) protein family, inhibiting their regulation of the G_1_/S-phase checkpoint of the cell cycle. As a result, viral infection stimulates cell cycle progression into S phase, facilitating viral DNA genome replication (5, 6). LTAg also binds p53, altering the regulation of both apoptosis and cell cycle progression (7). stAg modulates the phosphorylation of >300 cell cycle proteins and LTAg through interaction with protein phosphatase 2A (8, 9). The role of agnoprotein is less well understood although a wide range of activities have been proposed, including acting as a viroporin, enhancing viral DNA replication through interaction with the processivity factor proliferating cell nuclear antigen (PCNA), and enhancing egress of virions from the nucleus (10, 11).

The limited coding capacity of BKPyV necessitates co-option of multiple host factors in order to replicate and persist. Previous studies investigating how BKPyV infection modulates the host cell environment have primarily been conducted at the level of the transcriptome, which may not be reflected in the proteome. Infection in primary renal proximal tubule epithelial (RPTE) cells and human umbilical vein endothelial cells (HUVEC) has been studied using either microarray (12, 13) or transcriptome sequencing (RNA-seq) (14, 15). Such analyses do not provide information about virus-induced changes to cellular proteins. To date, there has been only one limited analysis of changes to the host cell proteome in BKPyV infection, where stable isotope labeling with amino acids in cell culture (SILAC) was used to quantify protein changes in nuclei isolated from primary RPTE cells at 3 days postinfection. In this study, ∼2,000 proteins were quantified, and the effect of infection on proteins outside the nucleus could not be assessed (16).

To gain a comprehensive global understanding of changes in host and viral proteins throughout the whole course of BKPyV infection, we conducted a 10-plex quantitative temporal viromic analysis (QTV) of two independent BKPyV-permissive primary human cell types, RPTE and human urothelial (HU) cells. QTV uses tandem mass tags (TMT) and MS3 mass spectrometry (MS/MS/MS) to quantify the relative abundances of proteins throughout the whole time course of infection (17). These data have provided the broadest global analysis of proteome changes caused by BKPyV infection, which has provided additional details of a specialized form of cell cycle arrest that is induced by this virus in primary cells. In addition, we have uncovered a complete lack of induction of innate immune responses at the protein level in BKPyV-infected cells, suggesting that this virus has evolved a sophisticated mechanism for evading pathogen recognition.

(This article was submitted to an online preprint archive [18]).

## RESULTS

### Quantitative temporal viromics analysis of BKPyV infection.

**FIG 1:**
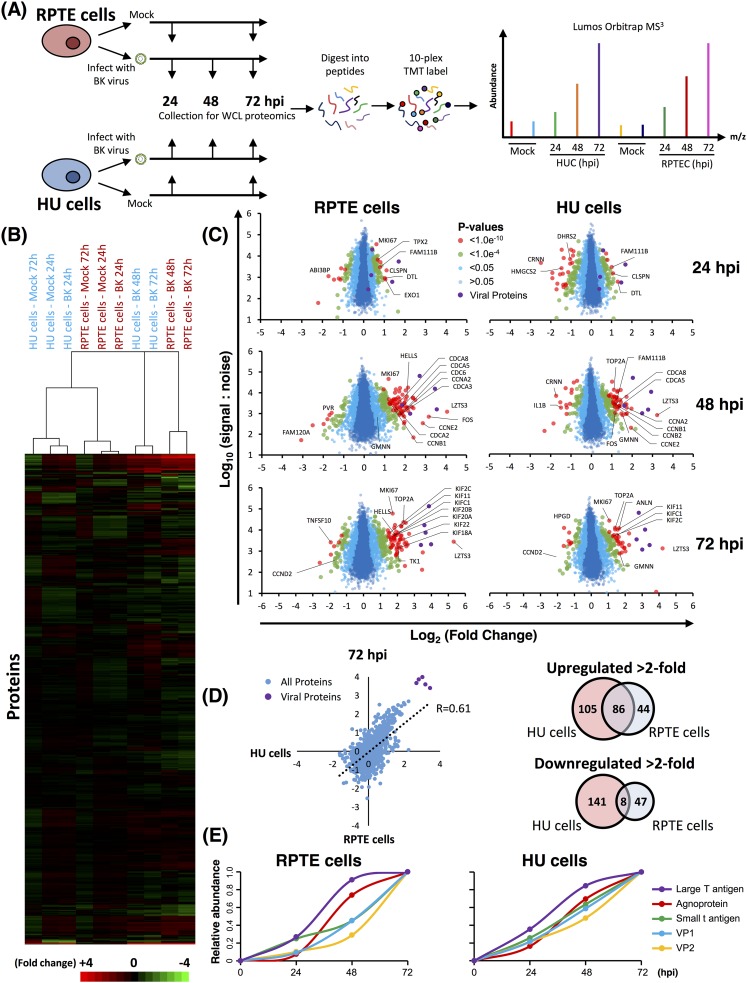
Quantitative temporal analysis of BK polyomavirus lytic infection. (A) Schematic of experimental workflow. RPTE and HU cells (RPTEC and HUC, respectively) were infected at an MOI of 5 or mock infected. Whole-cell lysates (WCL) were harvested at 24, 48, and 72 h (infected samples) or at 24 and 72 h (mock-infected samples). (B) Hierarchical cluster analysis of all quantified proteins. (C) Scatter plots of all proteins quantified at 24, 48, and 72 hpi in RPTE and HU cells. Fold change is shown in comparison to the average corresponding value of the mock infection for the same cell type. Benjamini-Hochberg-corrected significance B was used to estimate *P* values (51). (D) Scatter plot showing the correlation between protein abundance changes in BKPyV-infected RPTE and HU cells and overlap of proteins up- and downregulated by >2-fold between RPTE and HU cells. (E) Temporal profiles of the 5 viral proteins identified, normalized to a maximum of one.

To build a global picture of changes in host and viral proteins throughout the course of BKPyV infection, we infected primary renal (RPTE) and bladder (HU) epithelial cells with the BKPyV Dunlop strain. We first used 10-plex TMT and MS3 mass spectrometry to quantify changes in protein expression over three key time points of infection spanning the single-step replication cycle of this virus (0 to 72 h; experiment 1) (Fig. 1A). Cells were infected at a multiplicity of infection (MOI) of 5 infectious units per cell, ensuring greater than 90% infection in both RPTE and HU cells (data not shown). In this experiment a total of 8,985 cellular and 5/7 viral proteins were quantified in both cell types, providing a global view of changes in protein expression during infection in primary human epithelial cells from the kidney and bladder. Data from all proteomic experiments in this study are shown in Table S1 in the supplemental material, which includes a worksheet titled “Plots’’ that is interactive, enabling generation of graphs of protein expression of any of the human and viral proteins quantified.

In uninfected cells, RPTE and HU cells exhibit differential expression of proteins, as expected from two different cell types. In infected cells, few changes occurred by 24 h of infection; however, more substantial differences were seen by 48 and 72 h (Fig. 1B and C). In RPTE cells 191 cellular proteins increased >2-fold, while 149 proteins decreased >2-fold at any time point during BKPyV infection. In HU cells 130 proteins increased >2-fold and 55 decreased >2-fold. Many proteins showed similar changes in both cell types although cell-type-specific effects were also seen (Fig. 1D) (*R* = 0.61). We reasoned that the protein changes which were important for viral replication would be common to different cell types. By combining the two data sets, we found that just 86 cellular proteins, less than 1% of all proteins quantified, were upregulated >2-fold in both RPTE and HU cells (Fig. 1D).

**FIG 2:**
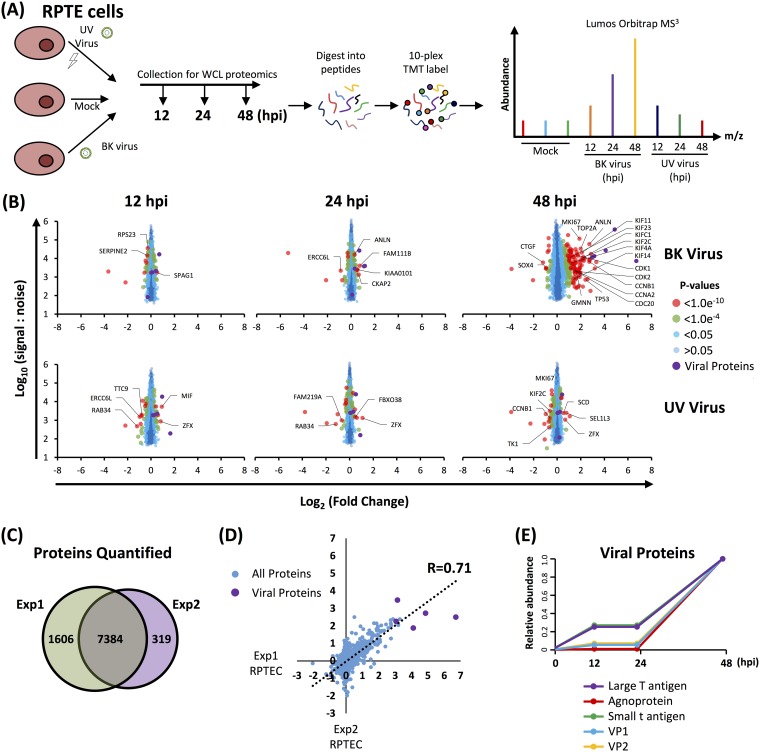
Repeat quantitative temporal profiling in RPTE cells (experiment 2). (A) Schematic of experimental workflow for experiment 2. (B) Scatter plots of all proteins quantified. Fold change is shown in comparison to the average corresponding value of the mock infection. Benjamini-Hochberg-corrected significance B was used to estimate *P* values (51). (C) Overlap of proteins quantified between experiment 1 and experiment 2. (D) Scatter plot showing the correlation between experiments 1 and 2 in RPTE cells for proteins quantified by ≥2 peptides. (E) Temporal profiles of the 5 viral proteins quantified, normalized to a maximum of 1.

The lack of change in the host cell proteome at the earliest time point of 24 h postinfection (hpi) suggested little or no effect of virus binding and penetration. To investigate this further, a second TMT-based whole-cell proteomics experiment (experiment 2) was conducted repeating 24- and 48-hpi time points with an additional earlier 12-hpi time point, where RPTE cells were infected with UV-inactivated or unmodified BKPyV at an MOI of 5 (Fig. 2 and Table S1). In experiment 2, a total of 7,698 cellular proteins were quantified, some of which were not detected in experiment 1, giving a combined total of 9,304 cellular proteins quantified across both experiments. Very few changes in protein abundance were observed at 12 or 24 hpi during infection with unmodified BKPyV, while at 48 hpi cellular proteins upregulated were similar to those observed in the first experiment at the same time point (Fig. 2B to D). UV-inactivated virus induced virtually no changes at any time point, suggesting that virus replication is necessary to cause the observed changes in host protein abundance (Fig. 2B).

### Temporal analysis of BK polyomavirus protein expression.

Expression of the early BKPyV proteins, LTAg and stAg, was observed from 24 hpi, closely followed by late proteins, agnoprotein, VP1, and VP2. Profiles from HU and RPTE cells (both experiments) corresponded well (Fig. 1E and 2E). We were unable to assign peptides to VP3 due to its 100% sequence identity with the C terminus of VP2, and the single unique peptide corresponding to the extreme N terminus of VP3 was not quantified. Likewise, truncTAg was not identified due to its similarity to full-length LTAg: the only difference in the protein sequences are the C-terminal 3 amino acids of truncTAg, which directly follow a cluster of lysine and arginine residues and so would not be expected to be identified by our mass spectrometry analysis.

### BKPyV does not cause induction of innate immune responses in infected RPTE cells.

**FIG 3:**
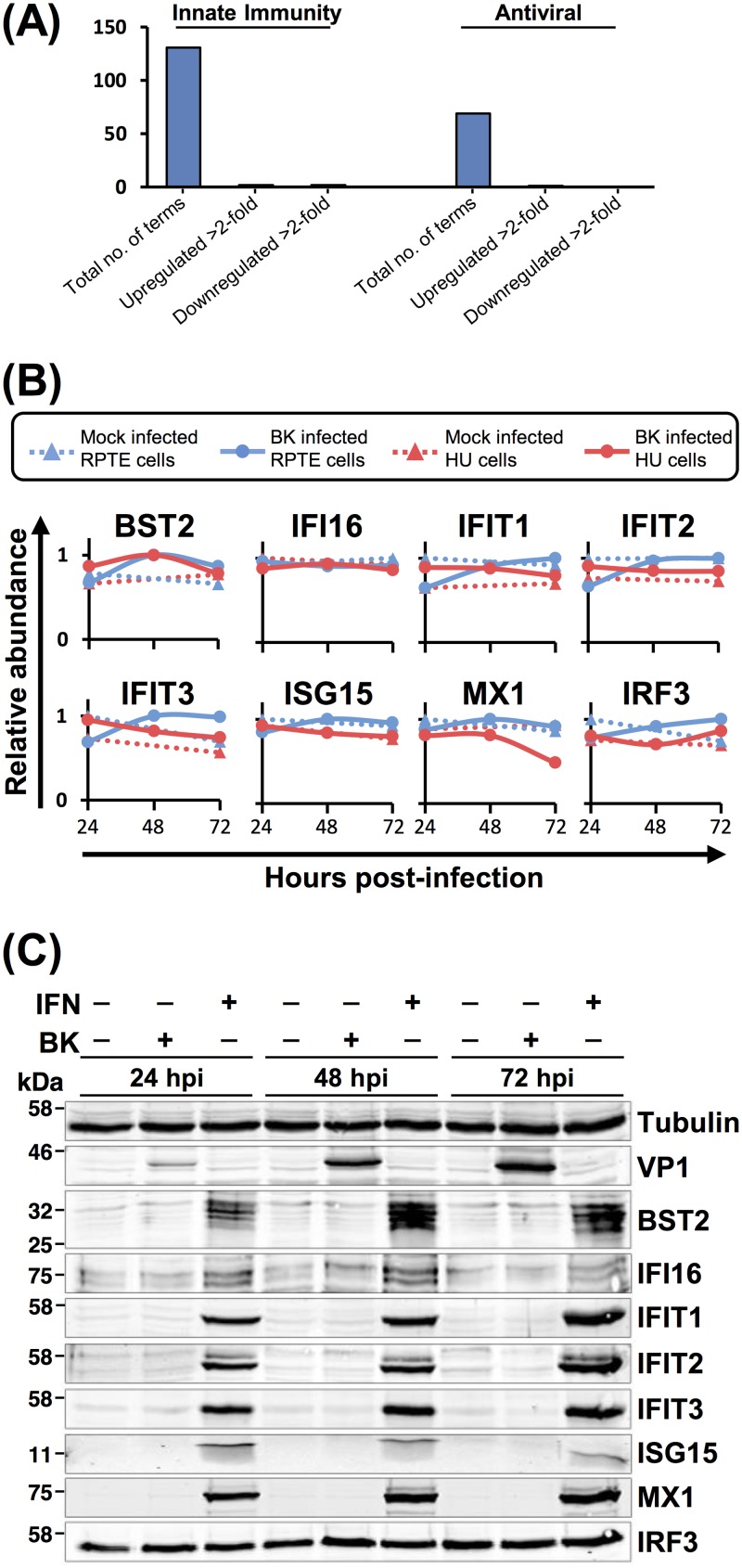
Proteins involved in the innate antiviral immune response remain unchanged during BKPyV infection. (A) Up- or downregulation of a minority of proteins with innate antiviral function (UniProt keywords, innate immunity and antiviral). (B) Example protein profiles from the data in panel A. (C) Validation of temporal profiles shown in panel B by Western blotting. RPTE cells were mock infected or infected with BKPyV at an MOI of 3 or stimulated with IFN-α2A (10^4^ U/ml) and analyzed by Western blotting for the proteins shown.

One surprising observation from our QTV analyses was an apparent lack of an innate immune response to BKPyV infection. Of the 131 quantified proteins with annotated innate immune functions or the 69 quantified proteins with annotated antiviral functions, only 5 were up- or downregulated >2-fold, and these changes were not consistent between the two independent cell types or experiments (Fig. 3A and Table S2). Even though RPTE cells are capable of mounting a response to type I interferon (IFN), the expression of interferon-stimulated genes (ISGs) MX1, ISG15, IFIT1, IFIT2, IFIT3, IRF3, IFI16, and BST2 remained unchanged upon BKPyV infection throughout the time course, as assessed both by proteomics and Western blotting (Fig. 3B and C). This was unexpected, given that by 72 hpi large amounts of viral DNA and proteins as well as progeny virions were present within cells. This lack of response suggests that BKPyV has evolved a highly effective immune evasion activity, which could be due to either viral DNA and proteins not being recognized by host pathogen recognition receptors (PRRs) in these primary epithelial cells or to suppression of PRR signaling pathways during BKPyV infection.

**FIG 4:**
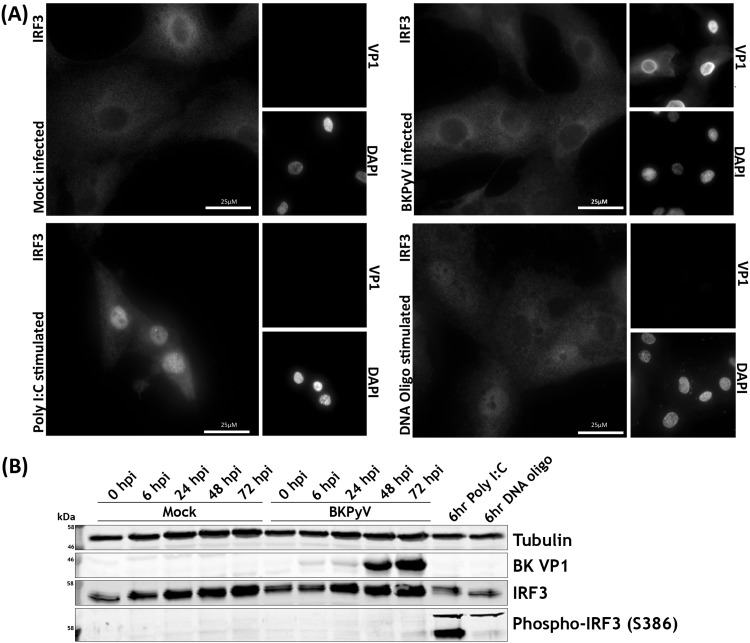
RPTE cells phosphorylate and translocate IRF3 in response to cytoplasmic RNA and DNA but fail to do so upon BKPyV infection. (A) Immunofluorescence microscopy analysis of IRF3 localization changes upon stimulation. RPTE cells infected with BKPyV (MOI of 0.5) or mock infected were fixed at 48 hpi. RPTE cells stimulated with poly(I·C) (2 μg/ml) or stimulatory DNA (2 μg/ml) were fixed at 6 h after stimulation. DAPI was used as a nuclear marker, and anti-VP1 was used as a marker of infection. (B) Analysis of IRF3 phosphorylation by Western blotting. RPTE cells were infected with BKPyV (MOI of 3) or mock infected and harvested at 48 hpi. RPTE cells stimulated with poly(I·C) (2 μg/ml) or stimulatory DNA (2 μg/ml) were harvested at 6 h after stimulation.

Activation of RNA and DNA sensors invariably leads to interferon regulatory factor 3 (IRF3) phosphorylation and translocation into the nucleus, leading to transcription of type I and III interferons. We analyzed whether RPTE cells have functional RNA and DNA sensing pathways and whether these were activated in response to BKPyV infection. The phosphorylation and localization of IRF3 were investigated by Western blotting and immunofluorescence microscopy following BKPyV infection or treatment with poly(I·C) or stimulatory DNA. Poly(I·C) or stimulatory DNA caused clear nuclear translocation of IRF3 in RPTE cells, with poly(I·C) having the greatest effect (Fig. 4A). However, BKPyV-infected RPTE cells had no detectable change in IRF3 localization and appeared no different from mock-infected cells apart from characteristic enlarged nuclei in virus-infected cells (Fig. 4A). Furthermore, Western blot analysis showed no stimulation of IRF3 phosphorylation in BKPyV-infected RPTE cells, whereas both poly(I·C) and stimulatory DNA transfection caused robust IRF3 phosphorylation (Fig. 4B). These results suggest that signal transduction pathways that would usually lead to activation of IRF3-specific kinases are not activated in infected cells due either to an inability to sense BKPyV nucleic acids or to active inhibition by BKPyV.

**FIG 5:**
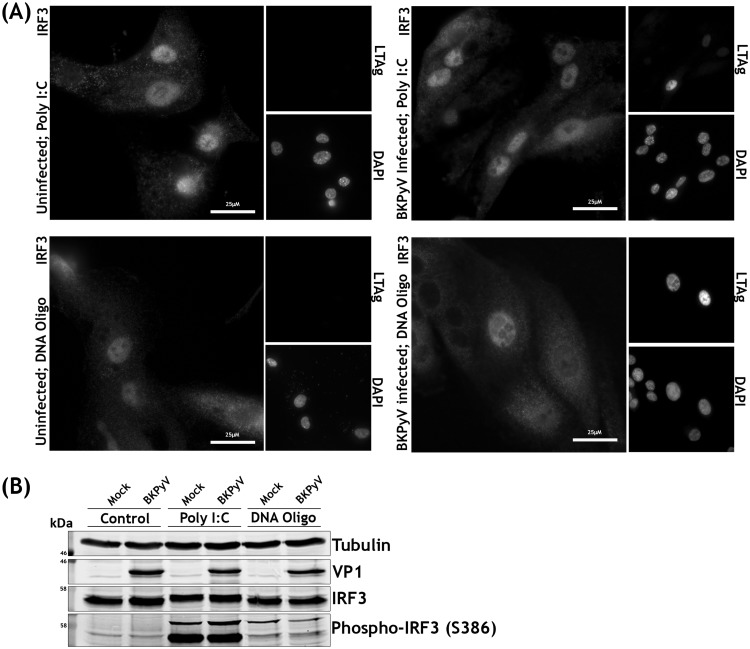
BKPyV and mock-infected RPTE cells do not differ in their responses to cytoplasmic RNA and DNA. (A) Immunofluorescence microscopy analysis of IRF3 localization changes upon stimulation in BKPyV-infected or uninfected cells. RPTE cells infected with BKPyV (MOI of 0.5) or mock infected were stimulated with poly(I·C) (2 μg/ml) or oligomeric DNA (2 μg/ml) at 42 hpi and fixed at 48 hpi. DAPI was used as a nuclear marker, and anti-LTAg was used as a marker of infection. (B) Analysis of IRF3 phosphorylation upon stimulation in BKPyV-infected or uninfected cells by Western blotting. RPTE cells infected with BKPyV (MOI of 3) or mock infected were stimulated with poly(I·C) (2 μg/ml) or oligomeric DNA (2 μg/ml) at 42 hpi and harvested at 48 hpi.

To investigate whether the lack of viral sensing is due to evasion of nucleic acid detection or active suppression of IRF3 phosphorylation, RPTE cells were mock or BKPyV infected and subsequently stimulated with poly(I·C) or stimulatory DNA at 42 hpi, prior to analysis at 48 hpi. Nuclear translocation and robust phosphorylation of IRF3 were observed in response to both RNA and DNA, irrespective of whether the cells were infected with BKPyV or mock infected (Fig. 5A and B). This suggests that BKPyV does not actively inhibit nucleic acid sensing pathways, IRF3 phosphorylation, or IRF3 nuclear translocation. As BKPyV does not inhibit downstream activation of RNA or DNA sensing pathways, this suggests that BKPyV evades nucleic acid and other pathogen-associated molecular pattern (PAMP) sensing pathways altogether, despite high concentrations of viral DNA, RNA, and protein within these primary renal epithelial cells.

### Cell cycle-associated proteins are the primary target of BKPyV.

**FIG 6:**
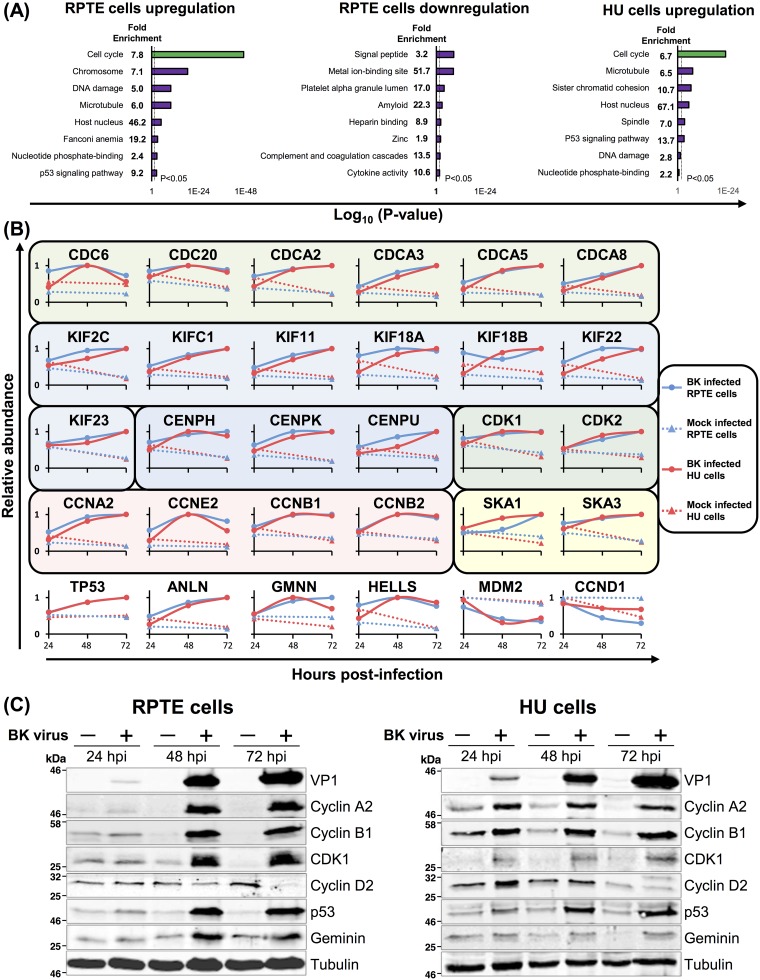
Upregulated proteins are enriched in cell cycle functions. (A) DAVID enrichment analysis of proteins upregulated or downregulated >2-fold against a background of all 8,985 human proteins quantified in experiment 1. No significantly enriched downregulated clusters were observed for HU cells. (B) Example protein profiles for selected cell cycle-related proteins for both RPTE and HU cells. Protein families are separated by colored boxes. (C) Validation of selected temporal profiles shown in panel B by Western blotting (RPTE cells; MOI of 3). Tubulin was used as a loading control, and VP1 was used as a control for infection.

To investigate host cell functions that were modified by BKPyV, the Database for Annotation, Visualization and Integrated Discovery (DAVID) was used to identify pathways enriched among proteins up- or downregulated during BKPyV infection (19). Among upregulated proteins, similar terms were enriched between HU and RPTE cells (from both experiments 1 and 2). Cell cycle and terms related to the cell cycle dominated this analysis (Fig. 6A and Tables S3 and S4). Terms associated with the G_2_/M phase of the cell cycle were particularly enriched, including the following: chromosome, microtubule, spindle, sister chromatid cohesion, and DNA damage. G_2_/M phase arrest has previously been observed in a number of different polyomavirus infections (20–23).

Cellular proteins associated with G_2_/M phase of the cell cycle generally increased in abundance throughout BKPyV infection, including the following: M phase (CDCA3), spindle formation (CDC20, CDCA2), kinetochore assembly, sister chromatid segregation and cytokinesis (KIF11, CENPK, SKA1, KIF22, and ANLN), DNA repair and control of rereplication (HELLS and GMNN), and G_2_/M-associated cyclins and cyclin-dependent kinases (CDK1, cyclin A2, and cyclin B1) (Fig. 6B). Proteins associated with the G_1_ phase, such as cyclin D2, were observed to decrease in abundance. As expected, levels of the tumor suppressor p53 were elevated during BKPyV infection; polyomavirus LTAg binds, stabilizes, and inactivates p53 (5, 24, 25). Interestingly, MDM2, the ubiquitin ligase that normally mediates p53 degradation, was depleted during BKPyV infection (Fig. 6B).

We confirmed these results for a number of cell cycle regulatory proteins by Western blotting throughout the time course of BKPyV infection in both RPTE and HU cells (Fig. 6C). Immunofluorescence microscopy of BKPyV or mock-infected RPTE cells further confirmed the increase in cyclin B1 and CDK1 during BKPyV infection and, furthermore, that cyclin B1 remained cytoplasmic during BKPyV infection (data not shown). This suggests that infected cells do not proceed into M phase when cyclin B1 would normally relocalize to the nucleus (26).

### MDM2 and p53 levels are modulated by LTAg and cell cycle arrest.

**FIG 7:**
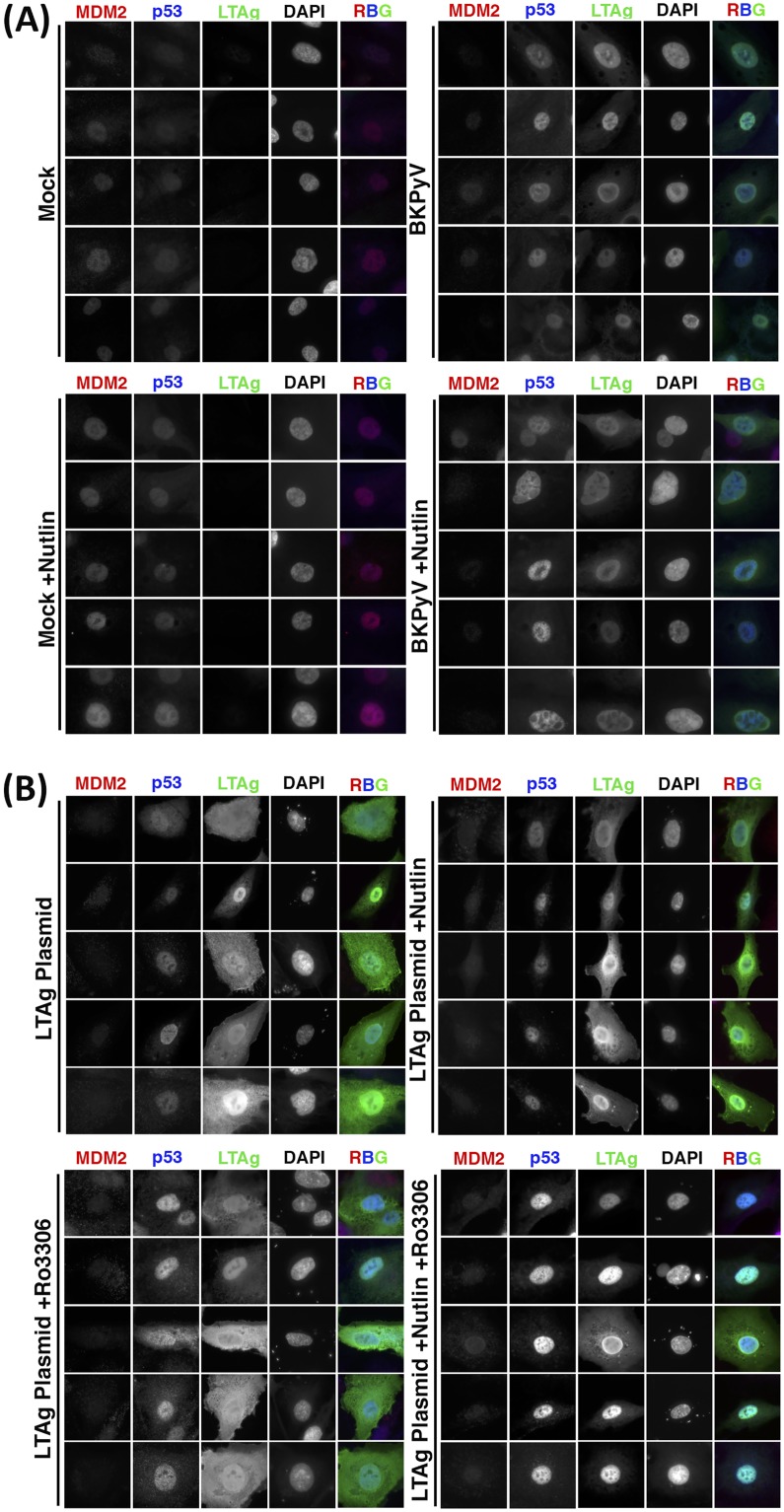
MDM2 and p53 are modulated by BKPyV via LTAg-dependent and -independent activities. (A) Expression of MDM2, p53, and LTAg in RPTE cells infected with BKPyV (MOI of 1) or mock infected, then treated with 5 μM Nutlin-3 or DMSO as a control at 2 hpi, and fixed at 48 hpi. DAPI was used as a nuclear marker. (B) Expression of MDM2, p53, and LTAg in RPTE cells transfected with BKPyV LTAg, then treated with 5 μM Nutlin-3 or DMSO as a control at 2 h and subjected to cell cycle inhibition (5 μM RO-3306) at 24 h, and fixed at 48 h posttransfection. DAPI was used as a nuclear marker. Five example cells are shown for each condition.

BKPyV-induced upregulation of p53 and downregulation of MDM2 were also confirmed by immunofluorescence (Fig. 7). The E3 ubiquitin ligase MDM2 is a negative regulator of both p53 and itself, leading to ubiquitinylation and degradation of p53 and MDM2 (27). In addition, p53 is a transcription factor for both itself and MDM2 (28), whose transcriptional activity is governed by the strength of extracellular and intracellular signals, such as cell cycle checkpoints, leading to the establishment of both positive- and negative-feedback loops (29).

Polyomavirus LTAgs are well established to efficiently bind, stabilize, and inhibit p53 (5, 24, 25), leading to increased p53 levels in BKPyV-infected cells. Our data now demonstrate that this is accompanied by a decrease in MDM2 levels. Interplay between BKPyV infection, MDM2, and p53 was investigated using the MDM2 inhibitor Nutlin-3. Nutlin-3 occupies the p53 binding pocket on MDM2, obstructing their interaction and leading to reduced p53 ubiquitinylation. In addition, Nutlin-3 leads to increased transcription of MDM2 due to the release of active p53 (30). Mock- or BKPyV-infected RPTE cells were treated with Nutlin-3 at 2 hpi or with dimethyl sulfoxide (DMSO) as a control and fixed at 48 hpi. Cells were immunostained for expression of MDM2, p53, and LTAg (infection marker) (Fig. 7A). Low endogenous levels of both MDM2 and p53 were observed in the nuclei of untreated mock-infected cells. BKPyV infection led to a reduction in MDM2, while p53 increased, correlating with the changes observed in the proteomics data. Mock-infected cells treated with Nutlin-3 showed increased levels of MDM2, accompanied by a slight increase in p53 levels, in accordance with published effects of Nutlin-3 (30). Interestingly, MDM2 levels did not increase in BKPyV-infected cells treated with Nutlin-3, and, in fact MDM2 levels were observed to decrease while p53 levels once again increased. This suggests that during infection MDM2 remains able to self-ubiquitinylate, leading to its degradation in the presence of Nutlin-3; however, transcription of MDM2 by p53 is apparently inhibited, likely due to p53 sequestration by LTAg.

To investigate whether LTAg expression alone was sufficient to cause the observed MDM2 decrease and p53 increase, RPTE cells were transfected with an LTAg expression plasmid. At 2 h cells were treated with Nutlin-3 or DMSO, and then in addition some samples were treated at 24 h with a CDK1-specific inhibitor, RO-3306, to simulate BKPyV-induced cell cycle arrest. Cells were fixed at 48 h and immunostained for MDM2, p53, and LTAg (Fig. 7B). Expression of LTAg alone was sufficient to reduce MDM2 levels; however, p53 levels were increased only slightly, suggesting that other effects of BKPyV infection in addition to LTAg expression modulate p53 and MDM2 levels. Nutlin-3 treatment did not alter the effects of LTAg on MDM2 or p53 levels. Treatment of LTAg-expressing cells with the CDK1 inhibitor RO-3306 led to a marked increase in p53 expression, while MDM2 levels were once again decreased. Combined Nutlin-3 and RO-3306 treatment further enhanced the increase of p53 in LTAg-expressing cells. Taken together, these data suggest that LTAg binding to p53 displaces MDM2, leading to p53 stabilization and MDM2 degradation, but LTAg binding also prevents p53-dependent expression of MDM2 and p53. Furthermore, virus infection or G_2_/M arrest stimulates p53 expression, possibly via a DNA damage-type response.

### BKPyV induced G_2_/M-phase arrest is prevented by inhibition of CDK1 and CDK2 but not by inhibition of CDK1 alone or CDK4 and CDK6.

**FIG 8:**
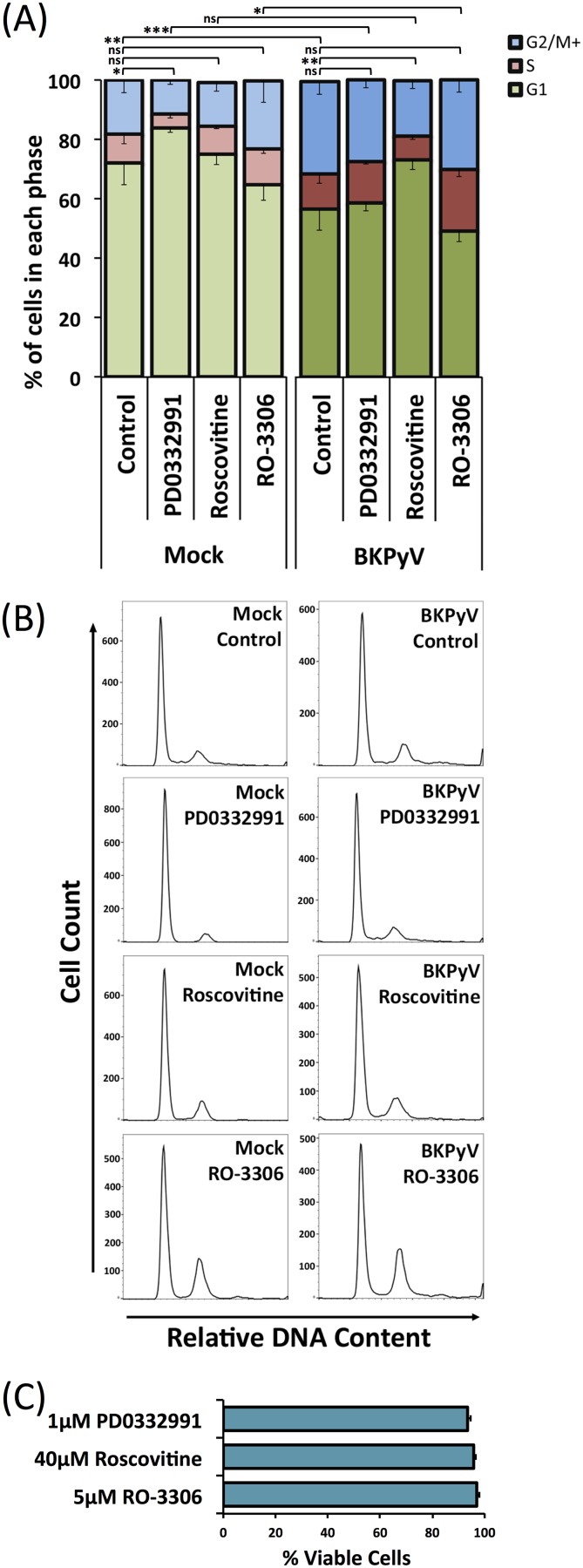
Cell cycle inhibitors have variable effects on BKPyV-induced G_2_/M phase cell cycle arrest. (A) The cell cycle status of RPTE cells was determined under a number of different experimental conditions. RPTE cells were infected with BKPyV (MOI of 3) or mock infected and then subjected to CDK4/6 inhibition (1 μM PD0332991), CDK1/2 inhibition (20 μM Roscovitine), or CDK1 inhibition (5 μM RO-3306) at 24 hpi; cells were subsequently collected for analysis at 48 hpi. Collected cells were stained with propidium iodide (PI) and analyzed by flow cytometry (*n* = 3). Error bars represent standard deviations. *P < 0.05; **P < 0.01; ***P < 0.001; ns, not significant (two-sample *t* test for changes in proportion of cells in G_1_). (B) Histograms of PI staining for each experimental condition of a single experiment are shown. (C) Cell viability tests. RPTE cells were treated with 1 μM PD0332991, 20 μM Roscovitine, or 5 μM RO-3306 for 24 h and then subjected to a trypan blue exclusion assay according to the manufacturer’s protocol (*n* = 3).

Given the dysregulation of cell cycle-related proteins during BKPyV replication, we postulated that a virus-induced pseudo-G_2_ phase may serve a number of roles in BKPyV replication. We therefore investigated the effect of BKPyV infection on the host cell cycle status in the presence or absence of various CDK inhibitors. Polyomavirus replication is heavily reliant on the host DNA synthesis machinery, and it has previously been shown that either BKPyV infection or JC polyomavirus (JCPyV) LTAg expression alone can cause cells to arrest in the G_2_/M phase of the cell cycle (23, 31, 32). However, the impact of CDK inhibitors on BKPyV-induced arrest has not been fully investigated. RPTE cells were infected with BKPyV, treated with CDK inhibitors at 24 hpi to allow sufficient time for virus entry and initiation of early gene expression, and then subsequently harvested at 48 hpi and analyzed by flow cytometry to compare cell cycle profiles. PD0332991 was used to inhibit CDK4 and -6 (CDK4/6), which are active in G_1_ phase; Roscovitine was used to inhibit CDK1 and 2 (CDK1/2), which are active throughout the S, G_2_, and M phases; and RO-3306 was used to inhibit CDK1, which is active in the G_2_ and M phases. In mock-infected RPTE cells, all three inhibitors produced the expected effects: PD0332991 increased the proportion of cells in G_1_ from 72% to 84% (*P* < 0.05), Roscovitine produced little change in the proportion of cells in any cell cycle phase because of its broad effect on S, G_2_ and M phases, and RO-3306 produced an increased proportion of cells in G_2_/M (18% to 23%) and reduced proportion in G_1_ (72% to 65%) although this did not reach statistical significance (Fig. 8A and B). Cell viability remained above 90% for all inhibitor conditions used (Fig. 8C). Infection of RPTE cells with BKPyV in the absence of any inhibitor significantly increased the proportion of cells in G_2_/M from 18% to 31% and decreased the proportion of cells in G_1_ (72% to 56%; *P* < 0.01), consistent with the BKPyV-induced G_2_ arrest observed in previously published data (31). BKPyV-infected cells that were treated with PD0332991 showed a significant decrease in the proportion of cells in G_1_ compared to that in uninfected cells treated with PD0332991 (84% to 58%; *P* < 0.001). While a slight increase was observed in the proportion of cells in G_1_ for BKPyV-infected and PD0332991-treated cells compared to levels in control BKPyV-infected cells (56% to 58%), this did not reach statistical significance. This suggests that inhibition of CDK4 and -6 does not prevent BKPyV driving infected cells through the G_1_/S checkpoint (due to Rb inactivation by LTAg) or arresting cells in G_2_/M. In contrast, treatment of infected cells with Roscovitine, which inhibits both CDK1 and -2, appears to severely restrict BKPyV-stimulated S-phase entry and G_2_/M arrest as the cell cycle status was similar to that of mock-infected cells, with no significant change in the proportions of cells in any cell cycle phase. BKPyV-infected cells treated with RO-3306 (CDK1 inhibitor) showed a cell cycle profile similar to that of control BKPyV-infected cells, with no significant difference in the proportions of cells in any cell cycle phase (Fig. 8A). Comparison of mock-infected RO-3306-treated cells with BKPyV-infected RO-3306-treated cells showed an increased proportion of cells in G_2_/M and S with a corresponding decrease in G_1_ (65% to 49%; *P* < 0.05). This suggests that BKPyV infection and CDK1 inhibition have similar and additive effects on the cell cycle, namely, induction of G_2_/M arrest.

### Inhibition of CDK1 and CDK2 or CDK1 alone reduces BKPyV replication.

**FIG 9:**
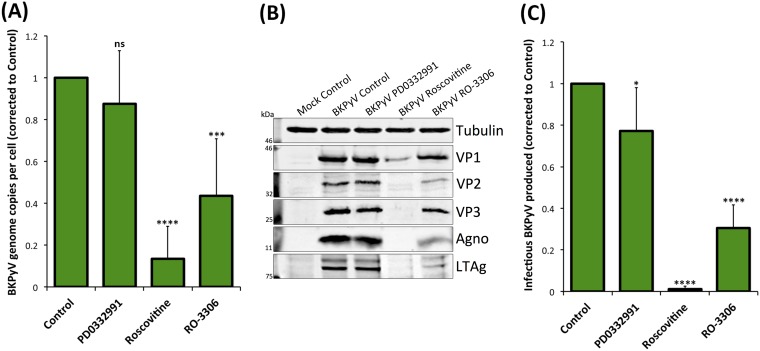
CDK1 and 2 inhibitors impede BKPyV replication in RPTE cells. RPTE cells infected with BKPyV (MOI of 3) were subjected to CDK4/6 inhibition (1 μM PD0332991), CDK1/2 inhibition (20 μM Roscovitine), or CDK1 inhibition (5 μM RO-3306) from 24 hpi and harvested for analysis at 48 hpi. (A) qPCR to determine viral DNA copy numbers per cell. DNA was extracted from each condition, BKPyV genome copy number was determined, normalized to that of the host gene (TNF-α) copy number, and compared to that of the uninhibited control, the value for which was arbitrarily set to 1 (*n* = 6). (B) Expression of viral proteins VP1, VP2, VP3, agnoprotein (Agno), and LTAg was determined by Western blotting. Tubulin was used as a loading control. (C) Infectious BKPyV produced under each experimental condition was determined by fluorescent focus unit (FFU) assay and normalized to the value of the uninhibited control (arbitrarily set to 1) (*n* = 7). Error bars represent standard deviations. *P < 0.05; ***P < 0.001; ****P < 0.0001; ns, not significant (one-sample *t* test experimental conditions versus control).

The ability of BKPyV to induce a pseudo-G_2_ arrest in the presence of CDK1 or CDK4/6 inhibition suggested that virus replication should be unaffected under such conditions while inhibition of CDK1/2 should perturb viral replication due to inhibition of S-phase progression. To investigate if this was the case, we next analyzed the effect of CDK inhibitors on viral genome synthesis in BKPyV-infected RPTE cells. Infected cells were treated with each inhibitor at 24 hpi and harvested at 48 hpi. Viral and host cell DNAs were quantified using quantitative PCR (qPCR) to determine viral DNA copy numbers per cell and were normalized to the level of uninhibited controls (arbitrarily set to 1). Inhibition of CDK4/6 had no significant effect on viral genome synthesis, while inhibition of CDK1 and -2 by Roscovitine showed a 7.4-fold reduction in the synthesis of BKPyV genome, likely due to the restriction of cells from entering and progressing through S phase (Fig. 9A). Surprisingly, inhibition of CDK1 alone by RO-3306 also caused a significant, although more modest, 2.3-fold reduction in BKPyV genome synthesis. This suggests that, despite this inhibitor having little effect on BKPyV-driven cell cycle progression, CDK1 activity is important for efficient viral genome synthesis.

The effects of these CDK inhibitors on viral protein synthesis was similarly investigated (Fig. 9B). Inhibition of CDK4 and -6 had no observable effect on viral protein synthesis while inhibition of CDK1 and -2 by Roscovitine substantially reduced viral protein expression levels. Inhibition of CDK1 alone by RO-3306 produced only small reductions in viral protein levels.

Analysis of infectious virus production in the presence or absence of these CDK inhibitors also demonstrated a similar trend. Inhibition of CDK4 and -6 caused only a slight reduction of infectious titers, whereas inhibition of CDK1 and -2 by Roscovitine resulted in a significant 80-fold reduction of virus production, unsurprisingly, given the inhibition of viral DNA and protein synthesis (Fig. 9C). Inhibition of CDK1 alone by RO-3306 caused a significant reduction of infectious virus titer by >3-fold. These data further suggest that CDK1 activity is important for the efficient production of infectious viruses.

## DISCUSSION

By employing the power and sensitivity of TMT-based MS3 mass spectrometry technology, we have been able to uncover BKPyV-induced changes to protein abundance and a global view of protein expression within human cells (HU and RPTE cells). Importantly, these studies were conducted in primary human cells from epithelial tissue representing the natural sites of replication *in vivo*. Therefore, this work also provides a comprehensive proteomic resource for future studies on human renourinary epithelial biology.

One of the most surprising findings of this study was just how few of the ∼9,000 cellular proteins that were quantified changed in abundance in response to BKPyV infection. In fact, just 235 were found to be upregulated and 196 were downregulated >2-fold or more across either cell type at any time point, which corresponds to <5% of the total proteome. Previous studies that applied a similar TMT-based approach to infection with human cytomegalovirus (HCMV), another dsDNA virus, revealed that 56% of cellular proteins changed in abundance more than 2-fold during the course of infection (17). This suggests that BKPyV and, presumably, other polyomaviruses are so highly adapted to their host that they need to induce only subtle changes to host gene expression to reprogram cells into virus-producing factories. This also suggests that polyomaviruses can very effectively evade detection by host pathogen recognition receptors despite producing high concentrations of foreign (viral) nucleic acid and proteins during productive infection.

For host proteins induced by BKPyV infection, we identified substantial overlap between the two primary cell types, with many of the same or highly related functional clusters identified by DAVID analysis. This includes clusters such as DNA damage and the Fanconi anemia pathway, which have been previously described as important during polyomavirus replication to ensure that viral genome replication maintains high fidelity (31). Interestingly, the majority of the functional clusters identified as upregulated in BKPyV infection are related to cell cycle activity and regulation, in particular, activities associated with G_2_ and M phases. In fact, BKPyV infection appears to have a G_2_/M arrest effect on cell cycle status similar to that of the CDK1-specific inhibitor RO-3306, a drug commonly used to arrest cells in G_2_. The fact that infected cells do not progress into authentic mitosis is supported by the observation that cyclin B1 remains predominantly cytoplasmic despite higher protein levels in infected cells and supports previously published data indicating that G_2_/M-phase arrest is driven by polyomavirus infection (5–7, 23, 33). Our data now provide a greater understanding of host protein profiles that are associated with polyomavirus-induced G_2_ arrest. It would be interesting to compare these observations to the effects of RO-3306 or other specific CDK1 inhibitors on cellular protein expression profiles.

**FIG 10:**
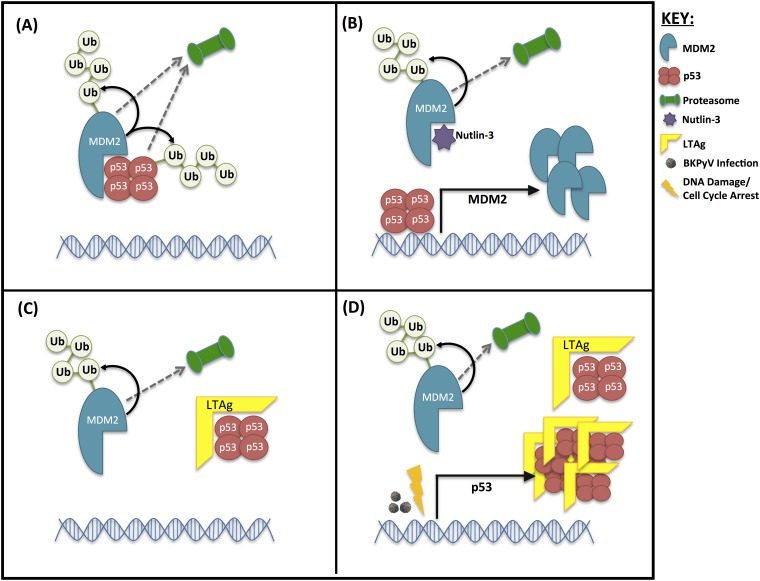
Proposed mechanism of interplay between MDM2 and p53 levels in the presence of LTAg. (A) Untreated cells. (B) Uninfected, untransfected cells inhibited with Nutlin-3. (C) Cells expressing LTAg in the absence of infection. (D) Cells infected with BKPyV or cells expressing LTAg combined with cell cycle arrest/DNA damage. Ub, ubiquitin.

Our data also indicate a specific perturbation of the p53-MDM2 axis by BKPyV infection, where MDM2 is reduced and p53 is increased but kept inactive by LTAg binding. However, these changes require more than just LTAg expression and are also driven by additional effects of BKPyV infection related to G_2_/M arrest and, potentially, DNA damage responses. Our findings suggest the following model: low MDM2 and p53 levels are maintained in uninfected cells due to their polyubiquitinylation by MDM2 and subsequent proteosomal degradation (Fig. 10A). Inhibition of MDM2-p53 interaction by Nutlin-3 releases p53, which then stimulates MDM2 expression (Fig. 10B). Interaction of LTAg with p53 displaces MDM2, thereby causing MDM2 to be destroyed by the proteasome, and so protects p53 from degradation but inhibits p53 transcriptional activity to prevent induction of *de novo* MDM2 expression (Fig. 10C). Therefore, the expression of just LTAg results in decreased MDM2 levels but only a modest increase in p53. During active BKPyV infection, p53 expression is induced by some other effect(s) of virus replication, and these additional copies of p53 are also bound and inactivated by LTAg (Fig. 10D). We predict that stimulation of p53 expression during BKPyV infection is via a DNA damage response pathway, which can be mimicked by inducing a G_2_ arrest through inhibition of CDK1.

Polyomaviruses have a well-established capacity to drive cells into S phase by overriding the G_1_/S checkpoint via the activity of LTAg. It is therefore unsurprising that inhibition of CDK4 and -6 has little to no effect on the ability of BKPyV to drive cell cycle progression or to replicate. CDK4 and -6, in complex with cyclin D, are normally responsible for phosphorylation of Rb and release of E2F proteins, allowing passage through the G_1_/S checkpoint (34). This is bypassed through the binding of LTAg to Rb family proteins, releasing E2F proteins enabling S-phase entry that is unconstrained by upstream factors (5, 6).

In contrast, Roscovitine, a potent inhibitor of CDK1 and -2, caused a global cell cycle arrest, irrespective of BKPyV infection, and reduced BKPyV replication. Similar effects of Roscovitine on polyomavirus replication have been previously been attributed to inhibition of CDK1 activity alone (35). However, our data suggest that the effects of Roscovitine are more likely due to inhibition of CDK2 activity or to the combination of inhibiting both CDK1 and -2. Inhibition of both of these cyclin-dependent kinases causes a rather global block to cell cycle progression; CDK2 is active in both late-G_1_ and S phases, while CDK1 is active in G_2_ and M phases (36). CDK2 activity is required immediately after G_1_ checkpoint clearance and beyond, and so the primary cause of BKPyV inhibition by Roscovitine could be due to a failure to activate S-phase proteins required for viral genome synthesis and consequent protein expression. Interestingly, we also observed reduced expression of LTAg in Roscovitine-treated cells. This might be attributed to inhibited progression through S phase, thus leading to reduced viral genome copy numbers from which LTAg is transcribed, although other effects of Roscovitine that inhibit transcription, such as inhibition of CDK7 and -9, may also contribute to this effect (37)

Somewhat more intriguing is the effect of CDK1-specific inhibition on BKPyV infection; RO-3306 caused significant reductions in viral DNA synthesis and infectious virus assembly. This was surprising because CDK1 activity is normally important for the transition through G_2_ and into M phase, and so inhibition of CDK1 would not be expected to inhibit progression through S phase and, thus, viral DNA replication. Whether CDK1 activity is required directly or indirectly to enhance DNA synthesis or other S-phase activities required for BKPyV genome replication or the process of virion assembly remains to be determined.

Moreover, our data have demonstrated that BKPyV infection of renourinary epithelial cells does not appear to cause the induction of antiviral responses, in agreement with published data of RPTE cells (12, 14, 15). Both RPTE and HU cells express the appropriate receptors, signaling pathways, and transcription factors associated with sensing and responding to DNA viruses, such as cyclic GMP-AMP synthase (cGAS; MB21D1), IFI16, STING (TMEM173), NF-κB, and IRF3, which were readily detected in our mass spectrometry analysis (see Table S1 in the supplemental material). RPTE cells are quite capable of responding to foreign intracellular DNA or RNA, leading to phosphorylation and nuclear translocation of IRF3, and RPTE cells also robustly express antiviral genes in response to type I interferon. However, we could not detect activation of these pathways even by 3 days after BKPyV infection: IRF3 remains unphosphorylated and cytoplasmic, and no ISGs were induced. Furthermore, we have also shown that active BKPyV infection within the same cell does not prevent the phosphorylation and nuclear translocation of IRF3 in response to either cytoplasmic RNA or DNA. This suggests that BKPyV is not actively suppressing such antiviral responses but, rather, prevents its own detection by pathogen recognition receptors. This evasion of detection may be due to a combination of having a small circular double-stranded DNA genome that is associated with histones, thus appearing similar to open chromatin, and tightly regulated entry and egress mechanisms to prevent exposure of viral DNA in the cytoplasm. Whether an inability to sense and respond to BKPyV infection is partly due to the nature of epithelial cells in the renourinary systems and whether this contributes to the natural tropism of BKPyV for these tissue types will be interesting questions for future study.

In summary, we have generated extensive data sets on the protein expression profiles of primary epithelial cells of the kidney and bladder using advanced multiplexed proteomics and provided a detailed understanding of how infection by BKPyV modifies the protein expression profiles in these cells. This research has provided additional details of the specific cell cycle arrest induced by virus infection and revealed the importance of this arrest for BKPyV replication. Furthermore, our findings suggest a surprising ability of BKPyV to evade detection and activation of innate immune responses in cells that are natural sites of lytic virus infection *in vivo*.

## MATERIALS AND METHODS

### Cell types, virus, and primary antibodies.

HU cells were grown in urothelial cell medium enriched with urothelial cell growth supplement and penicillin-streptomycin solution (Caltag Medsystems). HU cells were used at passages 4 to 6 for all experiments. RPTE cells were grown in renal epithelial basal medium enriched with an renal epithelial cell growth medium (REGM) BulletKit (Lonza). RPTE cells were used at passages 6 and 7 for all experiments.

BKPyV (Dunlop strain) inserted into pGEM7Zf(+) vector (kindly provided by M. Imperiale, University of Michigan) was digested with BamHI, purified, and religated. The resultant BKPyV-Dunlop genome was transfected into a T150 flask of RPTE cells; 1 week later the flask was split into three T150 flasks of RPTE cells. After a period of up to 4 weeks, virus was harvested by freeze-thawing cells three times. Virus purification by sucrose cushion, followed by cesium chloride gradient and dialysis, provided purified BKPyV stocks, as described previously (38). Concentration and purity were assessed by fluorescent focus unit (FFU) assay and Coomassie gel staining, respectively.

The primary antibodies used in this study were PAb597 against simian virus 40 (SV40) VP1 (kindly provided by W. Atwood, Brown University), P5G6 against BKPyV VP1 (kindly provided by D. Galloway, Fred Hutchinson Cancer Research Center), ab6160 against tubulin (Abcam), ab32386 against cyclin A2 (Abcam), ab32053 against cyclin B1 (Abcam), MA5-11472 against CDK1 (Thermo Scientific), ab207604 against cyclin D2, ab1101 against p53 (Abcam), GTX116125 against Geminin (GeneTex), ab16895 against MDM2 (Abcam), 37849 against MX1 (Cell Signaling Technologies), 2758 against ISG15 (Cell Signaling Technologies), PA3-848 against IFIT1 (ThermoFisher), 12604-1-AP against IFIT2 (ProteinTech), SAB1410691 against IFIT3 (Sigma-Aldrich), 11904 against IRF3 (Cell Signaling Technologies), ab76493 against IRF3 (phospho-S386) (Abcam), ac-8023 against IFI16 (Santa Cruz), 11721 against BST2 (NIH AIDS Reagent Program), ab53983 against SV40 VP2 and VP3 (Abcam), ab16879 against SV40 LTag (Abcam), and antibody against BKPyV agnoprotein (rabbit polyclonal antibody generated against agnoprotein-specific peptide).

### Cell infections and harvesting virus.

For viral infections, RPTE or HU cells were infected with BKPyV at either an MOI of 5 for TMT and validation experiments or an MOI of 3 or 0.5 for all other experiments, diluted in appropriate medium. At 1 hpi medium was removed, cells were washed twice with phosphate-buffered saline (PBS), and fresh medium was added. For TMT analysis, cells were harvested in TMT lysis buffer (6 M guanidine HCl, 50 mM HEPES, pH 8.5), vortexed extensively, and incubated at room temperature for 10 min. Lysates were then sonicated at 25 W for 30 s, followed by centrifugation at 21,000 × *g* for 10 min, after which supernatant was transferred to a fresh tube. Centrifugation was repeated, and supernatants were snap-frozen in liquid nitrogen for further processing. For Western blotting, cells were harvested by centrifugation at 6,000 × *g* after two PBS washes.

### Transfection.

RPTE cells were transfected with pcDNA3-LTAg plasmid using TransIT-LT1 transfection reagent (Mirus) in Opti-MEM medium according to the manufacturer’s protocol.

### Inhibitors.

For p53-MDM2 interaction inhibition experiments, cells were treated at 2 hpi with Nutlin-3. Nutlin-3 (Sigma) was made up to 20 mM in DMSO and used at 5 μM. For cell cycle inhibition experiments, cells were treated with inhibitors at 24 hpi. PD0332991 (Sigma) was made up to 5 mM in distilled H_2_O (dH_2_O) and used at 1 μM, Roscovitine (Sigma) was made up to 20 mM in DMSO and used at 20 μM, and RO-3306 (Sigma) was made up to 20 mM in DMSO and used at 5 μM. Controls were subjected to treatment with an equivalent amount of DMSO at the greatest volume of any inhibitor used. Cells were harvested in 1 ml of medium at 48 hpi and either pelleted by centrifugation at 6,000 × *g* for use in Western blotting or qPCR or frozen for FFU assay. For analysis by flow cytometry, cells were detached from wells by trypsin-EDTA treatment, centrifuged at 6,000 × *g*, washed in PBS, and fixed in 70% ice-cold ethanol.

### FFU assays and immunofluorescence microscopy.

Fluorescent focus unit (FFU) assays were used to determine the concentration of infectious virus in purified BKPyV stocks or experimental samples. RPTE cells were infected with sample dilutions, fixed at 48 hpi, and immunostained for VP1 expression as described in Evans et al. (39). For comparison of inhibitor effects, infectious BKPyV levels in cells under uninhibited conditions were arbitrarily set to 1, and those under inhibited conditions were corrected to this control level for 7 independent experiments. A one-sample *t* test was conducted to give *P* values (standard deviations are shown with error bars).

For immunofluorescence analysis, RPTE cells were fixed in 3% formaldehyde. Fixed cells were then permeabilized and quenched (50 mM NH_4_Cl and 0.1% Triton X-100 in PBS), blocked in PGAT (0.2% gelatin, 0.01% Triton X-100, 0.02% NaN_3_ in PBS), and stained with primary antibodies. Secondary antibodies used for immunofluorescence were Alexa Fluor 568-conjugated donkey anti-mouse or goat anti-IgG1 mouse and Alexa Fluor 488-conjugated donkey anti-rabbit or goat anti-IgG2a mouse. Coverslips were mounted using SlowFade Gold with 4′,6′-diamidino-2-phenylindole (DAPI) (Invitrogen). Samples were imaged using a 63× oil immersion lens on an Olympus IX81 wide-field fluorescence microscope.

### Western blotting.

RPTE cells were lysed by suspension in modified radioimmunoprecipitation assay (mRIPA) buffer (50 mM Tris, pH 7.5, 150 mM NaCl, 1% sodium deoxycholate and 1% Triton X-100) supplemented with Complete protease inhibitors without EDTA (Roche). Cellular debris was removed by centrifugation at 17,000 × *g*. HU cells were lysed by suspension in HU cell lysis buffer (20 mM HEPES, pH 7.6, 250 mM sucrose, 2 mM dithiothreitol [DTT], 2 mM EDTA Na_2_, and 2 mM EGTA) supplemented with Complete protease inhibitors without EDTA, followed by sonication at 25 W for 30 s. Proteins were separated by SDS-PAGE and transferred to nitrocellulose membranes before being blocked in 5% skimmed milk powder in PBS.

Following primary antibody binding, Li-Cor IRDye 680-conjugated (anti-mouse, anti-rabbit, or anti-rat) or IRDye 800-conjugated (anti-mouse or anti-rabbit) secondary antibodies were used. Membranes were then imaged on a Li-Cor Odyssey infrared imaging system.

### Real-time PCR (qPCR).

RPTE cell pellets were lysed in 200 μl of NDA lysis buffer (4 M guanidine thiocyanate, 25 mM Tris, and 134 mM β-mercaptoethanol) and incubated at 56°C for 10 min, after which an equal volume of 100% ethanol was added. DNA was then bound to silica columns by centrifuging at 16,000 × *g* for 1 min. Columns were washed with buffer 1 (1 M guanidine thiocyanate, 25 mM Tris, pH 7, in 10% ethanol) and centrifuged, followed by a final wash in buffer 2 (25 mM Tris, pH 7, in 70% ethanol). DNA was eluted with nuclease-free water by centrifugation at 16,000 × *g*. Primers and probe for the BKPyV genome were designed as described in Evans et al. (39). Human tumor necrosis factor alpha (TNF-α) primers and probe were designed and obtained through TIB MolBiol (forward primer, AGGAACAGCACAGGCCTTAGTG; reverse primer, AAGACCCCTCCCAGATAGATGG; TaqMan probe, CCAGGATGTGGAGAGTGAACCGACATG). A 300 nM concentration of each primer and 50 nM TaqMan probe were used in each qPCR reaction mixture, which was run on a Rotor-Gene instrument (RG-3000; Corbett Research) and subsequently analyzed on Rotor-Gene software. BKPyV genome levels were corrected to the level of the TNF-α control for each sample, and values of uninhibited samples were arbitrarily set to 1 (6 independent experiments). A one-sample *t* test was conducted to give *P* values (standard deviations are shown with error bars).

### Flow cytometry.

Cellular DNA content was used as an indicator of cell cycle status. Cells were fixed in 70% ethanol for 30 min, DNA was stained by resuspending each PBS-washed cell pellet in 0.2 mg of RNase A and 50 μg of propidium iodide (PI) in 1 ml of PBS and incubated at 37°C for 1 h. Cells were then centrifuged at 6,000 × *g*; supernatant was removed, and cells were resuspended in 500 μl of PBS. Cells were analyzed by flow cytometry using a BD FACSCantoII with BD FACSDiva software (BD Biosciences) and further analyzed using the FlowJo, version 10.4.2, cell cycle analysis function. A minimum of 10,000 cells were collected for each sample (3 independent experiments). Two-tailed Student’s *t* tests were conducted to assess significance of changes in cell cycle status between samples. Standard deviation error was calculated for each cell cycle status sample.

### Whole-cell lysate protein digestion.

Cells were washed twice with PBS, and 250 μl of lysis buffer was added (6 M guanidine, 50 mM HEPES, pH 8.5). Cell lifters (Corning) were used to scrape cells in lysis buffer, which was removed to an Eppendorf tube, vortexed extensively, and then sonicated. Cell debris was removed by centrifugation at 21,000 × *g* for 10 min twice. Dithiothreitol (DTT) was added to a final concentration of 5 mM, and samples were incubated for 20 min. Cysteines were alkylated with 14 mM iodoacetamide and incubated for 20 min at room temperature in the dark. Excess iodoacetamide was quenched with DTT for 15 min. Samples were diluted with 200 mM HEPES, pH 8.5, to 1.5 M guanidine, followed by digestion at room temperature for 3 h with LysC protease at a 1:100 protease/protein ratio. Samples were further diluted with 200 mM HEPES, pH 8.5, to 0.5 M guanidine. Trypsin was then added at a 1:100 protease/protein ratio, followed by overnight incubation at 37°C. The reaction was quenched with 5% formic acid (FA) and then centrifuged at 21,000 × *g* for 10 min to remove undigested protein. Peptides were subjected to C_18_ solid-phase extraction (SPE) (Sep-Pak, Waters) and vacuum centrifuged to near dryness.

### Peptide labeling with tandem mass tags.

In preparation for TMT labeling, desalted peptides were dissolved in 200 mM HEPES, pH 8.5. Peptide concentration was measured by a Micro BCA (bicinchoninic acid) assay (Pierce), and 25 μg was labeled with TMT reagent. TMT reagents (0.8 mg) were dissolved in 43 μl of anhydrous acetonitrile, and 3 μl was added to peptides at a final acetonitrile concentration of 30% (vol/vol). Samples were prepared with TMT labels as follows for experiment 1 (10-plex); TMT-126, HU cells in mock infection at 24 hpi; 127N, HU cells in mock infection at 72 hpi; 127C, HU cells in BKPyV infection 24 hpi; 128N, HU cells BKPyV infection 48 hpi; 128C, HU cells in BKPyV infection at 72 hpi; 129N, RPTE cells in mock infection at 24 hpi; 129C, RPTE cells in mock infection at 72 hpi; 130N, – RPTE cells in BKPyV infection at 24 hpi; 130C, RPTE cells in BKPyV infection at 48 hpi; 131N, RPTE cells in BKPyV infection 72 hpi. Samples were prepared with TMT labels as follows for experiment 2 (9-plex); TMT-126, mock infection 12 hpi; 127N, mock infection 24 hpi; 127C, mock infection at 48 hpi; 128N, BKPyV infection at 12 hpi; 128C, BKPyV infection at 24 hpi; 129N, – BKPyV irradiated at 48 hpi; 129C – BKPyV irradiated at 12 hpi; 130N, BKPyV irradiated at 24 hpi; 130C, BKPyV infection at 48 hpi. Following incubation at room temperature for 1 h, the reaction was quenched with hydroxylamine to a final concentration of 0.3% (vol/vol). TMT-labeled samples were combined at a 1:1:1:1:1:1:1:1:1:1 ratio for experiment 1 and at a 1:1:1:1:1:1:1:1:1 ratio for experiment 2. The sample was vacuum centrifuged to near dryness and subjected to C_18_ SPE (Sep-Pak, Waters). An unfractionated single shot was initially analyzed to ensure similar peptide loading across each TMT channel to avoid the need for excessive electronic normalization. Quantities of each TMT-labeled sample were adjusted prior to high-pH reversed-phase (HpRP) fractionation so that normalization factors were >0.67 and <1.5. Normalization is discussed in the paragraph “Data analysis,” and fractionation is discussed below.

### Offline HpRP fractionation.

TMT-labeled tryptic peptides were subjected to HpRP fractionation using an Ultimate 3000 RSLC ultra-high-performance liquid chromatography (UHPLC) system (Thermo Fisher Scientific) equipped with a Kinetix Evo C_18_ column (internal diameter [i.d.], 2.1 mm; length, 25 cm; particle size, 1.7 μm) (Phenomenex). The mobile phase consisted of A (3% acetonitrile [MeCN]), B (MeCN), and C (200 mM ammonium formate, pH 10). Isocratic conditions were 90% A and 10% C, and C was maintained at 10% throughout the gradient elution. Separations were conducted at 45°C. Samples were loaded at 200 μl/min for 5 min. The flow rate was then increased to 400 μl/min over 5 min, after which the gradient elution proceeded as follows: 0 to 19% B over 10 min, 19 to 34% B over 14.25 min, and 34 to 50% B over 8.75 min, followed by a 10-min wash at 90% B. UV absorbance was monitored at 280 nm, and 15-s fractions were collected into 96-well microplates using an integrated fraction collector. Fractions were recombined orthogonally in a checkerboard fashion, combining alternate wells from each column of the plate into a single fraction and commencing combination of adjacent fractions in alternating rows. Wells prior to the start or after the stop of elution of peptide-rich fractions, as identified from the UV trace, were excluded. This yielded two sets of 12 combined fractions, A and B, which were dried in a vacuum centrifuge and resuspended in 10 μl of MS solvent (4% MeCN–5% formic acid) prior to liquid chromatography (LC)-MS3. Eleven set A fractions were used for experiment 1, and 12 set A and 9 set B fractions were used for experiment 2.

### LC-MS3.

Mass spectrometry data were acquired using an Orbitrap Lumos (Thermo Fisher Scientific, San Jose, CA). An Ultimate 3000 RSLC nano-UHPLC equipped with an Acclaim PepMap μ-Precolumn (300-μm i.d. by 5 mm; Thermo Fisher Scientific) and an Acclaim PepMap RSLC analytical column (75-μm i.d. by 50 cm; particle size, 2.1 μm) was used.

Loading solvent was 0.1% formic acid (FA); analytical solvent A consisted of 0.1% FA, and B consisted of 80% MeCN–0.1% FA. All separations were carried out at 55°C. Samples were loaded at 5 μl/min for 5 min in loading solvent before the analytical gradient was begun. The following gradient was used: 3 to 7% B over 3 min and 7 to 37% B over 173 min, followed by a 4-min wash at 95% B and equilibration at 3% B for 15 min. Each analysis used a MultiNotch MS3-based TMT method (40, 41). The following settings were used: MS1: 380-1500 Th, 120,000 resolution, 2 × 10^5^ automatic gain control (AGC) target, 50-ms maximum injection time. For tandem mass spectrometry (MS2), settings were as follows: quadrupole isolation at an isolation width of *m/z* 0.7, collision-induced dissociation (CID) fragmentation (normalized collision energy [NCE], 35) with ion trap scanning in turbo mode from *m/z* 120, 1.5 × 10^4^ AGC target, 120-ms maximum injection time. For MS3, in synchronous precursor selection mode, the top six MS2 ions were selected for high-energy collision dissociation (HCD) fragmentation (NCE, 65) and scanned in the Orbitrap at 60,000 resolution with an AGC target of 1 × 10^5^ and a maximum accumulation time of 150 ms. Ions were not accumulated for all parallelizable times. The entire MS/MS/MS cycle had a target time of 3 s. Dynamic exclusion was set to ±10 ppm for 70 s. MS2 fragmentation was trigged on precursors with 5 × 10^3^ counts and above.

### Data analysis.

In the following description, we list the first report in the literature for each relevant algorithm. Mass spectra were processed using a Sequest-based software pipeline for quantitative proteomics, MassPike, through a collaborative arrangement with Steve Gygi’s laboratory at Harvard Medical School. MS spectra were converted to mzXML using an extractor built upon Thermo Fisher’s RAW File Reader library (version 4.0.26). In this extractor, the standard mzxml format has been augmented with additional custom fields that are specific to ion trap and Orbitrap mass spectrometry and essential for TMT quantitation. These additional fields include ion injection times for each scan, Fourier transform-derived baseline and noise values calculated for every Orbitrap scan, isolation widths for each scan type, scan event numbers, and elapsed scan times. This software is a component of the MassPike software platform and is licensed by Harvard Medical School.

A combined database was constructed from (i) the human UniProt database (4 February 2014) and (ii) the BK polyomavirus database (6 October 2014). The combined database was concatenated with a reverse database composed of all protein sequences in reversed order. Searches were performed using a 20-ppm precursor ion tolerance (42). Product ion tolerance was set to 0.03 Th. TMT tags on lysine residues and peptide N termini (229.162932 Da) and carbamidomethylation of cysteine residues (57.02146 Da) were set as static modifications, while oxidation of methionine residues (15.99492 Da) was set as a variable modification.

To control the fraction of erroneous protein identifications, a target-decoy strategy was employed (43, 44). Peptide spectral matches (PSMs) were filtered to an initial peptide-level false discovery rate (FDR) of 1%, with subsequent filtering to attain a final protein-level FDR of 1% (45, 46). PSM filtering was performed using a linear discriminant analysis, as described previously (47). This distinguishes correct from incorrect peptide identifications in a manner analogous to the widely used Percolator algorithm (48) though employing a distinct machine learning algorithm. The following parameters were considered: XCorr, ΔCn, missed cleavages, peptide length, charge state, and precursor mass accuracy. Protein assembly was guided by principles of parsimony to produce the smallest set of proteins necessary to account for all observed peptides (47).

Proteins were quantified by summing TMT reporter ion counts across all matching peptide-spectral matches using MassPike, as described previously (40, 41). Briefly, a 0.003 Th window around the theoretical *m/z* of each reporter ion (ion channels 126, 127N, 127C, 128N, 128C, 129N, 129C, 130N, 130C, 131N, and 131C) was scanned for ions, and the maximum intensity nearest to the theoretical *m/z* was used. The primary determinant of quantitation quality is the number of TMT reporter ions detected in each MS3 spectrum, which is directly proportional to the signal-to-noise (S/N) ratio observed for each ion (49). Conservatively, every individual peptide used for quantitation was required to contribute sufficient TMT reporter ions (minimum of ∼1,250 per spectrum) so that each on its own could be expected to provide a representative picture of relative protein abundance (40). An isolation specificity filter was additionally employed to minimize peptide coisolation (50). Peptide-spectral matches with poor-quality MS3 spectra (more than 9 TMT channels missing and/or a combined S/N ratio of less than 250 across all TMT reporter ions) or no MS3 spectra at all were excluded from quantitation. Peptides meeting the stated criteria for reliable quantitation were then summed by parent protein, in effect weighting the contributions of individual peptides to the total protein signal based on their individual TMT reporter ion yields. Protein quantitation values were exported for further analysis in Excel.

For protein quantitation, reverse and contaminant proteins were removed, and then each reporter ion channel was summed across all quantified proteins and normalized assuming equal protein loading across all channels. For further analysis and display in figures, fractional TMT signals were used (i.e., reporting the fraction of maximal signal observed for each protein in each TMT channel rather than the absolute normalized signal intensity). This effectively corrected for differences in the numbers of peptides observed per protein. For all TMT experiments, normalized S/N values are presented in Table S1 in the supplemental material (data worksheet).

Hierarchical centroid clustering based on uncentered Pearson correlation, and *k*-means clustering were performed using Cluster, version 3.0 (Stanford University), and visualized using Java Treeview (http://jtreeview.sourceforge.net) unless otherwise noted.

### Data availability.

The mass spectrometry proteomics data have been deposited with the ProteomeXchange Consortium (http://www.proteomexchange.org/) via the PRIDE partner repository under the data set identifier PXD013940 (http://www.ebi.ac.uk/pride/archive/projects/PXD013940).

## Supplementary Material

Supplemental file 1

Supplemental file 2

Supplemental file 3

Supplemental file 4

Supplemental file 5
