# Supplementary material for: Temporal Proteomic Analysis of BK Polyomavirus Infection Reveals Virus-Induced G2 Arrest and Highly Effective Evasion of Innate Immune Sensing
Source: J Virol. 2019 Jul 30;93(16):e00595-19. doi: 10.1128/JVI.00595-19 (PMC6675895; doi:10.1128/JVI.00595-19)
Supplement: Supplemental file 5 [file JVI.00595-19-s0005.pdf]

Supplementary Data Table descriptions

Table S1. Interactive spreadsheet of all mass spectrometry data. The “DATA” worksheet shows minimally annotated protein data, with only formatting and normalisation modifying the raw data. The “Plotter” worksheet enables generation of individual protein abundance changes for both viral and human proteins over time from experiments 1 and 2. The total number of quantified peptides and proteins from experiments 1 and 2 are shown in separate workbook sheets.

Table S2. A list of all quantified proteins from experiments 1 and 2 that match the UniProt keywords terms ‘Innate Immunity’ and ‘Antiviral Defense’ and their corresponding fold changes post-BKPyV infection.

Table S3. Proteins modulated during BK infection. All proteins downregulated or upregulated >2-fold at any time point during the course of infection (compared to the average of the mock samples). Results from experiments 1 and 2 are presented.

Table S4. DAVID functional enrichment analysis from proteins upregulated or downregulated >2-fold against a background of all proteins quantified. Only significant (Benjamini-Hochberg corrected) clusters from experiments 1 and 2 are shown. There were no significant clusters amongst proteins downregulated >2-fold in experiment 2.
